# Thirty-Day Mortality After Hip Fracture Surgery: Association with In-Hospital Adverse Events and Comparative Performance of Comorbidity Indices

**DOI:** 10.3390/healthcare14131870

**Published:** 2026-06-26

**Authors:** Ana Šarić Jadrijev, Ana Maria Mitar, Ana Bego, Marija Jukica, Borna Lojpur, Dino Poljak, Grgur Prižmić, Vesna Čapkun, Katarina Vukojević, Petar Đolonga, Toni Kljaković-Gašpić, Nikola Delić, Andre Bratanić

**Affiliations:** 1 Department of Anaesthesiology, University Hospital of Split, Spinčićeva 1, 21000 Split, Croatia; asaric@kbsplit.hr (A.Š.J.);; 2 School of Medicine, University of Split, Šoltanska 2A, 21000 Split, Croatia; 3 Department of Psychiatry, General Hospital Šibenik, Ul. Stjepana Radića 83, 22000 Šibenik, Croatia; 4 Department of Surgery, Division of Orthopaedics and Traumatology, University Hospital of Split, Spinčićeva 1, 21000 Split, Croatia; 5 Department of Anatomy, Histology and Embryology, University of Split School of Medicine, Šoltanska 2A, 21000 Split, Croatia; 6 Department of Pathology, Judicial Medicine, and Cytology, University Hospital of Split, Spinčićeva 1, 21000 Split, Croatia; 7 Department of Internal Medicine, Division of Gastroenterology, University Hospital of Split, Spinčićeva 1, 21000 Split, Croatia

**Keywords:** hip fractures, mortality, in-hospital adverse events, comorbidity, ASA PS classification, Charlson Comorbidity Index, Elixhauser Comorbidity Index, frailty, aged

## Abstract

**Highlights:**

**What are the main findings?**

- In-hospital adverse events were associated with higher 30-day mortality after hip fracture surgery.
- Baseline-only models, based on the ASA PS Classification, the Charlson Comorbidity Index, and the Elixhauser Comorbidity Index, showed broadly comparable discrimination for 30-day mortality in this cohort.

**What are the implications of the main findings?**

- Routinely available comorbidity indices provided broadly similar short-term risk stratification, although their discrimination was acceptable rather than perfect.
- In-hospital adverse events should be interpreted as time-dependent clinical events associated with mortality rather than baseline predictors or evidence of causality.

**Abstract:**

**Background/Objectives**: Hip fractures are associated with high short-term mortality in older adults. This study aimed to determine 30-day mortality after hip fracture surgery and evaluate factors associated with short-term mortality, with particular attention to baseline comorbidity indices and in-hospital adverse events. **Methods**: This retrospective cohort study included 785 patients who underwent surgery for hip fracture at University Hospital of Split, Croatia, between January 2021 and December 2022. Clinical data were extracted from medical records. The primary outcome was 30-day mortality, including in-hospital and post-discharge deaths. Associations with mortality were examined using univariable and multivariable logistic regression. Baseline-only comorbidity models were constructed using the American Society of Anesthesiologists Physical Status Classification System (ASA PS Classification), the Charlson Comorbidity Index (CCI), and the Elixhauser Comorbidity Index (ECI). Exploratory hospital-course models additionally included in-hospital adverse events, which were interpreted as time-dependent hospital-course events rather than baseline predictors. **Results**: 30-day mortality was 11.0% (86/785). Older age, male sex, higher comorbidity burden, and in-hospital adverse events were associated with mortality. Mortality was 5.7% without documented adverse events, 24.2% with one adverse event, and 42.4% with two or more adverse events. Baseline-only comorbidity models showed acceptable and broadly comparable discrimination, with AUCs of 0.73–0.77. Exploratory hospital-course models showed higher discrimination, with AUCs of 0.80–0.82. **Conclusions**: 30-day mortality after hip fracture surgery was associated with baseline patient vulnerability and in-hospital adverse events. Baseline-only models based on the ASA PS Classification, the CCI, and the ECI provided broadly comparable short-term risk stratification. In-hospital adverse events should be viewed as markers of an adverse clinical trajectory, not evidence of causality.

## 1. Introduction

Hip fractures are a major cause of morbidity, functional decline, institutionalization, and mortality among older adults. With population aging, the burden of hip fractures is expected to increase, creating a growing challenge for healthcare systems [1,2,3,4]. Short-term mortality after hip fracture surgery remains substantial, commonly ranging from 8% to 15%, particularly among older patients with multiple comorbidities and limited physiological reserve [1,2,3,4,5,6].

Early mortality after hip fracture surgery is multifactorial. Advanced age, comorbidity burden, anemia, frailty, cognitive impairment, reduced pre-fracture mobility, and limited functional independence may all decrease physiological reserve and increase vulnerability to postoperative deterioration [3,4,5,6,7,8,9]. Frailty is especially relevant in this population because it reflects diminished capacity to respond to acute stressors and may influence both the development of in-hospital adverse events and the likelihood of short-term death [7,8,9].

Several routinely available clinical indices are used to summarize baseline health status and perioperative risk. These include the American Society of Anesthesiologists Physical Status Classification System (ASA PS Classification), the Charlson Comorbidity Index (CCI), and the Elixhauser Comorbidity Index (ECI) [10,11,12,13,14,15,16]. Although these indices capture overlapping aspects of comorbidity burden, they differ in complexity, clinical availability, and method of calculation. Previous studies have evaluated these indices in hip fracture populations [10,11,12]. However, less is known about how these three routinely available comorbidity indices perform side by side for 30-day mortality in regional real-world surgical cohorts, and how in-hospital adverse events should be interpreted alongside baseline comorbidity burden.

In addition to baseline vulnerability, events occurring during the hospital course may identify patients with an adverse clinical trajectory. Medical adverse events such as respiratory infections, urinary tract infections, venous thromboembolism, pressure ulcers, cardiovascular events, acute kidney injury, and neurocognitive adverse events have been associated with worse outcomes after hip fracture surgery. Reported rates vary across studies because of differences in patient case mix, event definitions, documentation practices, and follow-up duration [17,18,19,20,21]. Moreover, these events differ conceptually from baseline predictors because they occur any time after admission to hospital. Their interpretation requires caution because patients who die early may have less opportunity to develop or have documented adverse events, creating a potential survival or time-related bias [22].

The objective of this study was to determine 30-day mortality after hip fracture surgery in a contemporary regional cohort and to evaluate factors associated with short-term mortality. We specifically aimed to compare the discriminative performance of ASA PS Classification, CCI, and ECI for 30-day mortality and describe the associations between in-hospital adverse events and mortality. We framed in-hospital adverse events as time-dependent clinical events associated with mortality, rather than baseline predictors or evidence of causality.

## 2. Materials and Methods

### 2.1. Study Design and Setting

This retrospective cohort study was conducted at University Hospital Split, Croatia, a tertiary center providing specialized trauma and orthopedic care. Adult patients diagnosed with hip fractures (ICD-10 codes S72, S72.0, S72.1, and S72.2) and treated at the Department of Surgery between 1 January 2021, and 31 December 2022, were screened for eligibility. Patients were identified using the hospital information system (HIS). Clinical data were collected retrospectively from electronic medical records, patient charts, and anesthesia documentation archived at the Department of Surgery. The study was conducted and reported in accordance with the Strengthening the Reporting of Observational Studies in Epidemiology guidelines (STROBE) for cohort studies [23].

### 2.2. Study Population

**Figure 1:**
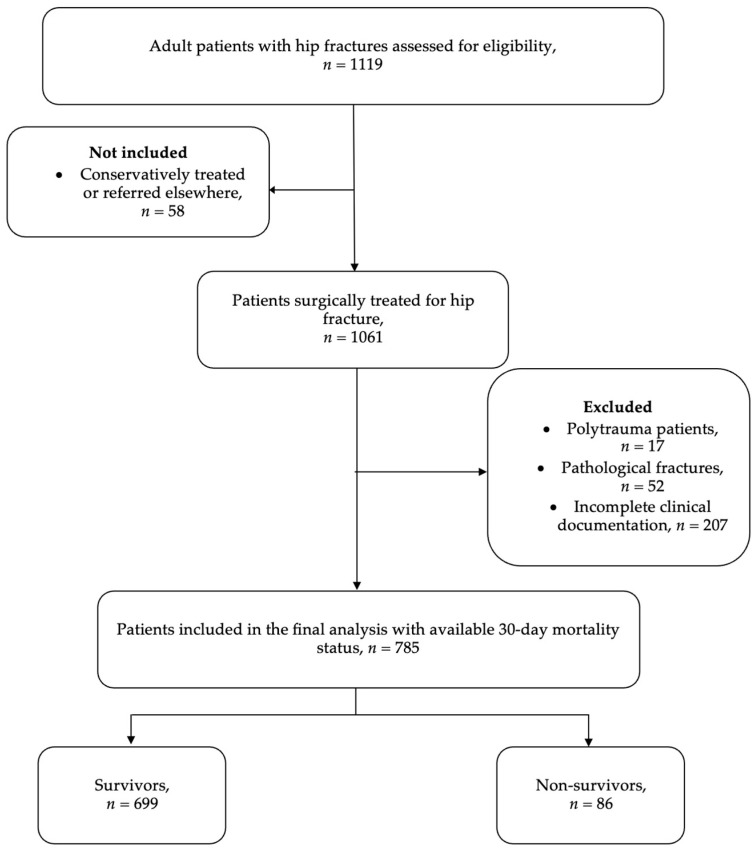
STROBE flow diagram of patient selection and 30-day follow-up.

All adult patients admitted with a diagnosis of hip fracture during the study period were evaluated for eligibility. During the study period, 1119 patients with hip fractures were identified. Hip fractures included intracapsular fractures (femoral neck fractures), extracapsular fractures (pertrochanteric and subtrochanteric fractures), and other surgically treated proximal femoral fracture patterns, including periprosthetic femoral fractures. Only patients who underwent surgical treatment were included in further analysis (*n* = 1061). A total of 58 patients were conservatively treated or referred elsewhere. Patients were excluded if they had incomplete clinical documentation (*n* = 207), had pathological fractures (*n* = 52), or were polytraumatized (*n* = 17). After application of the predefined inclusion and exclusion criteria, 785 surgically treated patients were included in the final study cohort. The patient selection process is shown in Figure 1.

Although protocol records were available for identifying eligible patients and creating flow charts, they did not contain a dataset for a formal comparison between included and excluded patients, which was therefore not performed. This limitation was taken into account when interpreting the findings.

### 2.3. Data Collection

Demographic, clinical, laboratory, and perioperative data were extracted from hospital medical records. Demographic variables included age and sex. Age was summarized descriptively as median and interquartile range and was additionally categorized into predefined groups approximating decade-based intervals for descriptive and exploratory stratification. Age category was entered as an ordinal variable in regression models, with higher categories representing older age.

Clinical variables included the ASA PS Classification System, the age-adjusted CCI, and the ECI. The CCI was calculated according to the original Charlson method with age adjustment. The ECI was calculated as a mortality-weighted Elixhauser score using the van Walraven point system, as recorded in the dataset from the Orthotoolkit Elixhauser comorbidity score calculator, available at https://orthotoolkit.com/elixhauser-comorbidity-index/ (accessed on 23 June 2026). Laboratory variables included admission hemoglobin and hematocrit levels. Information regarding pre-admission antithrombotic therapy was collected and categorized as none, acetylsalicylic acid, warfarin, direct oral anticoagulants (DOAC), or other therapy.

Perioperative variables included fracture type, type of surgical procedure, type of anesthesia, time to surgery, intensive care unit (ICU) length of stay, total length of stay, and perioperative transfusion. Time to surgery was expressed as day-level timing, as opposed to hour-level timing because exact time in hours was not consistently available in the retrospective documentation. This limits interpretation relative to guideline thresholds commonly expressed as 24–48 h.

In-hospital adverse events were defined as clinically documented adverse events occurring during the index hospitalization and recorded in the medical documentation or discharge summary. These events were grouped into clinically related non-surgical medical categories to facilitate interpretation, while recognizing that broad categories may include clinically heterogeneous conditions. Recorded in-hospital adverse events included respiratory infections, urinary tract infection, venous thromboembolism, cardiovascular events, pressure ulcer, acute kidney injury, and neurological adverse events including postoperative cognitive dysfunction. Respiratory infections and other medical adverse events were additionally separated into available subcomponents for descriptive reporting. Surgery-related adverse events, including surgical site infection, implant failure, reoperation, bleeding adverse events, and fixation-related problems, were not consistently available in the dataset and were therefore not analyzed.

### 2.4. Outcome Definition

The primary outcome was all-cause mortality within 30 days of the date of surgery. Mortality was defined as death occurring within 30 days of surgical treatment, including both in-hospital deaths and deaths occurring after hospital discharge. In-hospital deaths were recorded directly in the HIS. Post-discharge deaths were ascertained through the HIS, which is integrated with the Central Health Information System of the Republic of Croatia (CEZIH). In Croatia, all deaths must be reported to the relevant registry office within three days of occurrence and are subsequently entered into the national mortality database, ensuring near-complete capture of post-discharge mortality. Complete 30-day mortality status was confirmed for all 785 patients included in the final analysis.

Secondary outcomes included evaluation of clinical factors associated with 30-day mortality, the performance of comorbidity-based models, and the distribution of documented in-hospital adverse events.

### 2.5. Statistical Analysis

Distribution of continuous variables was assessed using the Shapiro–Wilk test together with visual inspection of distributions. Because continuous variables were not normally distributed, they were presented as medians with interquartile ranges and compared using the Mann–Whitney U test. Categorical variables were presented as numbers and percentages and compared using the chi-square test or Fisher exact test, as appropriate.

Univariable logistic regression analysis was performed to evaluate associations between clinical variables and 30-day mortality. Variables were selected for multivariable modelling using a combination of clinical relevance, avoidance of conceptual overlap, and univariable screening. Age, sex, comorbidity burden, admission hemoglobin, time to surgery, type of anesthesia, preoperative transfusion, and in-hospital adverse events were considered clinically relevant based on previous literature and clinical plausibility. Variables with *p* < 0.10 in univariable analysis were additionally considered to avoid excluding potentially relevant covariates at an early stage. Given 86 deaths, the number of covariates in each multivariable model was deliberately limited to reduce the risk of overfitting.

Three baseline-only multivariable logistic regression models were constructed to evaluate baseline short-term risk stratification using the ASA PS Classification, the age-adjusted CCI, and the ECI. The ASA PS baseline model included age category, sex, ASA PS Classification, and admission hemoglobin. The Charlson baseline model included sex, age-adjusted CCI, and admission hemoglobin. The Elixhauser baseline model included age category, sex, and ECI. These models were used as the primary baseline comorbidity model comparison.

Exploratory hospital-course multivariable models additionally included documented in-hospital adverse events. These models were constructed to describe associations observed during the index hospitalization and not to estimate baseline prediction or causal effects. Separate models were constructed using the ASA PS Classification, the age-adjusted CCI, and the ECI to avoid collinearity and conceptual overlap between composite indices. Age was not included as a separate covariate in the Charlson model because the age-adjusted CCI already incorporates age. Admission hemoglobin was not included in the Elixhauser model because anemia-related conditions were included among Elixhauser comorbidity measures, which could introduce conceptual overlap. The models were compared primarily for overall model performance and risk stratification, rather than direct comparison of individual covariate odds ratios across models.

In-hospital adverse events were included only in exploratory hospital-course models as events documented during the index hospitalization and associated with 30-day mortality. Because these events occurred any time after admission to hospital and were conditional on survival time, they were not interpreted as baseline predictors or evidence of causality. Exact dates of onset for all adverse events were not consistently available, preventing a valid time-dependent Cox model or landmark analysis. Therefore, the association between in-hospital adverse events and mortality was interpreted cautiously, purely as an indication of an adverse clinical trajectory. Analyses were performed as complete-case analyses after application of the predefined exclusion criteria; no multiple imputation was performed.

Model discrimination was evaluated using receiver operating characteristic curves (ROC) and expressed as the area under the curve (AUC) with bootstrap 95% confidence intervals. Calibration was assessed using the Hosmer–Lemeshow goodness-of-fit test, calibration plots, and the Brier score. Decision-curve analysis was performed as an exploratory assessment of net benefit across threshold probabilities from 0.01 to 0.50. These metrics were reported to strengthen transparent internal model evaluation, although the models were not externally validated and were not developed as definitive clinical prediction tools. Multicollinearity was assessed using variance inflation factors after inclusion of the intercept term. Because exact time to surgery in hours was not available, an additional descriptive day-level sensitivity analysis categorized time to surgery as 0–1 day, 2 days, and >2 days.

A two-sided *p*-value < 0.05 was considered statistically significant. Statistical analyses were performed using IBM SPSS Statistics for Windows, version 26.0 (IBM Corp., Armonk, NY, USA), with supplementary model-performance analyses performed in Python version 3.13.5 (Python Software Foundation, Beaverton, OR, USA), using NumPy version 2.3.5, pandas version 2.2.3, Matplotlib version 3.10.8, and scikit-learn version 1.8.0. Linearity of the logit for variables modeled as continuous or ordinal was assessed using exploratory spline-based likelihood-ratio checks comparing models with linear terms to models including spline terms for the same variables.

### 2.6. Ethical Approval

The study protocol was approved by the Ethics Committee of University Hospital Split (protocol code 500-03/22-01/21; approved on 3 March 2022). Due to the retrospective study design and the use of anonymized clinical data, the requirement for individual informed consent was waived.

## 3. Results

### 3.1. Demographic and Baseline Clinical Characteristics

A total of 785 surgically treated patients were included in the final analysis. The median age was 84 years (IQR 78–89), and 589 patients (75.0%) were female. ASA PS III-IV classification was recorded in 436 patients (55.5%), CCI ≥ 5 in 458 patients (58.3%), and ECI ≥ 5 in 367 patients (46.8%). No chronic antithrombotic therapy prior to admission was recorded in 529 patients (67.4%). Baseline and perioperative characteristics are presented in Table 1.

### 3.2. In-Hospital Adverse Events

Overall, 190 of 785 patients (24.2%) developed at least one documented in-hospital adverse event during the index hospitalization. The main adverse-event groups and corresponding 30-day mortality rates are presented in Table 2. Detailed components of broader adverse-event groups are provided in Supplementary Table S4.

### 3.3. Distribution of 30-Day Mortality According to Baseline Characteristics

Thirty-day mortality was 11.0% (86 of 785). Compared with survivors, non-survivors had a higher proportion of older age categories, male sex, ASA PS III-IV, CCI ≥ 5, and ECI ≥ 5. These baseline comparisons are presented in Supplementary Table S2.

### 3.4. Comparison of Continuous Clinical Variables by 30-Day Mortality Status

Compared with survivors, non-survivors had lower admission hemoglobin and hematocrit values, longer time to surgery, and longer ICU stay. These comparisons are presented in Supplementary Table S3. A descriptive day-level sensitivity analysis of time to surgery is presented in Supplementary Table S5.

### 3.5. Univariable Factors Associated with 30-Day Mortality

In univariable logistic regression analyses, older age category, male sex, comorbidity burden, lower admission hemoglobin, longer time to surgery, preoperative transfusion, general anesthesia, and the occurrence of any in-hospital adverse event were associated with 30-day mortality. Results are presented in Table 3.

### 3.6. Baseline-Only and Exploratory Hospital-Course Multivariable Models

Baseline-only models were used to evaluate baseline short-term risk stratification. In the baseline-only ASA PS model, older age category (OR 1.94, 95% CI 1.43–2.63), male sex (OR 3.58, 95% CI 2.16–5.94), and ASA PS Classification (OR 2.37, 95% CI 1.46–3.84) were associated with 30-day mortality, whereas admission hemoglobin was not statistically significant (OR 0.99, 95% CI 0.98–1.00). In the baseline-only Charlson model, male sex (OR 3.14, 95% CI 1.93–5.13), age-adjusted CCI (OR 1.34, 95% CI 1.20–1.50), and admission hemoglobin (OR 0.98, 95% CI 0.97–1.00) were associated with mortality. In the baseline-only Elixhauser model, older age category (OR 2.22, 95% CI 1.64–3.01), male sex (OR 3.59, 95% CI 2.18–5.91), and ECI (OR 1.07, 95% CI 1.04–1.11) were associated with mortality. Results of the baseline-only models are presented in Table 4.

In the exploratory ASA PS model, older age category (OR 2.01, 95% CI 1.46–2.76), male sex (OR 3.43, 95% CI 2.00–5.88), ASA PS Classification (OR 2.11, 95% CI 1.27–3.52), and any in-hospital adverse event (OR 4.86, 95% CI 2.90–8.15) were associated with mortality. In the exploratory Charlson model, male sex (OR 2.95, 95% CI 1.75–4.97), age-adjusted CCI (OR 1.33, 95% CI 1.18–1.50), and any in-hospital adverse event (OR 4.93, 95% CI 2.96–8.22) were associated with mortality. In the exploratory Elixhauser model, older age category (OR 2.14, 95% CI 1.56–2.94), male sex (OR 3.56, 95% CI 2.09–6.05), ECI (OR 1.07, 95% CI 1.03–1.11), and any in-hospital adverse event (OR 4.89, 95% CI 2.92–8.17) were associated with mortality. Time to surgery and preoperative transfusion were not associated with mortality after adjustment. General anesthesia was associated with mortality in the Charlson and Elixhauser exploratory models, but not in the ASA PS exploratory model. Because in-hospital adverse events occurred some point during hospitalization, odds ratios for this variable should not be interpreted as baseline risk estimates or causal effects. Results of the exploratory hospital-course models are presented in Table 5.

### 3.7. Model Performance

All three baseline-only comorbidity models, excluding in-hospital adverse events showed acceptable discrimination for 30-day mortality. The baseline-only ASA PS model had an AUC of 0.76 (95% CI 0.71–0.81), the baseline-only Charlson model had an AUC of 0.73 (95% CI 0.67–0.78), and the baseline-only Elixhauser model had an AUC of 0.77 (95% CI 0.71–0.82). Calibration was acceptable according to the Hosmer–Lemeshow test, and Brier scores were similar across baseline-only models.

**Figure 2:**
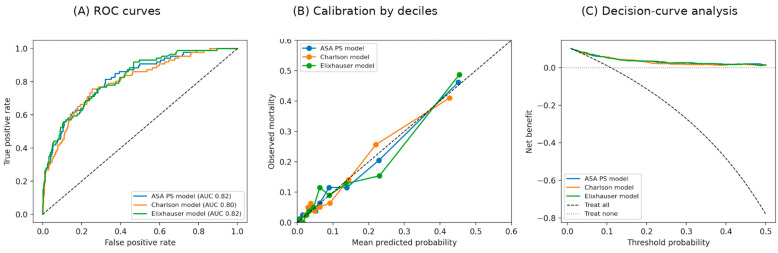
Predictive performance of the three exploratory hospital-course multivariable models for 30-day mortality. (**A**) Receiver operating characteristic curves; (**B**) calibration plots; and (**C**) decision-curve analysis. The three models were constructed using the ASA PS Classification, Charlson Comorbidity Index, and Elixhauser Comorbidity Index, respectively, and additionally included documented in-hospital adverse events. In panel (**A**), the dashed diagonal line represents the line of no discrimination. In panel (**B**), the dashed line represents ideal calibration. In panel (**C**), the dashed reference lines represent the treat-all and treat-none strategies.

Exploratory hospital-course models that additionally included in-hospital adverse events showed higher discrimination, with AUCs ranging from 0.80 to 0.82. However, because these models incorporate events documented during hospitalization, they should not be interpreted as baseline prediction models. Rather, they describe the combined association of baseline vulnerability and hospital-course adverse events with 30-day mortality. Model performance metrics for both baseline-only and exploratory hospital-course models are presented in Table 6. Predictive performance plots for the exploratory hospital-course models are shown in Figure 2.

### 3.8. In-Hospital Adverse Events and 30-Day Mortality

Mortality differed between patients with and without documented in-hospital adverse events and increased according to the number of documented events. These results are presented in Table 7.

## 4. Discussion

In this retrospective cohort of 785 surgically treated patients with hip fracture, 30-day mortality was 11.0%. Mortality was associated with baseline patient characteristics, including older age, male sex, and comorbidity burden, as well as with documented in-hospital adverse events. Baseline-only models, based on the ASA PS Classification, the CCI, and the ECI, showed acceptable and broadly comparable discrimination for 30-day mortality. Exploratory hospital-course models, including in-hospital adverse events, showed higher discrimination, but these models should not be interpreted as baseline prediction models. Importantly, in-hospital adverse events were interpreted as time-dependent clinical events associated with mortality and markers of an adverse clinical trajectory, rather than baseline predictors or causal determinants of death.

The observed 30-day mortality rate is consistent with contemporary hip fracture literature, which generally reports substantial short-term mortality after surgery [1,2,3,4,5,6]. Mortality after hip fracture is rarely attributable to a single factor. It reflects an interaction between baseline vulnerability, comorbidity burden, low admission hemoglobin, perioperative stress, frailty, and postoperative clinical deterioration [3,4,5,6,7,8,9,24]. In our cohort, older age and higher comorbidity burden were consistently associated with mortality, supporting the importance of baseline physiological reserve in early postoperative outcomes.

Frailty may help explain the observed associations. Frail patients have reduced capacity to compensate for acute illness, surgery, immobility, infection, and other postoperative stressors [7,8,9]. Although formal frailty measures were not available in this retrospective dataset, frailty could partly explain why patients with in-hospital adverse events had substantially higher mortality. In this context, adverse events should not be used to represent independent causes of mortality; rather, they might identify patients who were already on a high-risk trajectory due to underlying vulnerability, reduced functional reserve, or unmeasured geriatric factors.

In-hospital adverse events were strongly associated with 30-day mortality. Rather than indicating a direct causal effect, these events likely reflect the combined impact of baseline vulnerability, frailty, reduced physiological reserve, and acute postoperative deterioration. This interpretation is consistent with previous studies linking medical adverse events after hip fracture with worse outcomes [17,18,19,20,21]. Therefore, documented adverse events should be viewed primarily as clinical warning signals that may support closer monitoring, timely reassessment, and individualized postoperative care. Nevertheless, patients who died early may have had less opportunity to develop or have documented adverse events, which could introduce time-related bias [22].

Respiratory infections and pressure ulcers showed particularly high mortality in this cohort. These adverse events might reflect immobility, frailty, impaired pulmonary reserve, aspiration risk, poor nutritional status, or prolonged hospitalization. Urinary tract infections were the most frequent documented event but had lower observed mortality than respiratory infections and pressure ulcers. Rare events such as cardiovascular adverse events and venous thromboembolism were too infrequent for stable inference; estimates for these categories should therefore be interpreted descriptively.

The baseline-only models showed acceptable but not perfect discrimination for 30-day mortality, with broadly similar performance across the ASA PS Classification, age-adjusted CCI, and ECI. Exploratory hospital-course models that additionally included in-hospital adverse events showed higher discrimination than baseline-only models, but their performance was also broadly similar across the three comorbidity-index specifications. This suggests that the routinely available comorbidity measures captured overlapping aspects of baseline health status in hip fracture patients, while the addition of in-hospital adverse events reflected hospital-course clinical deterioration rather than improved baseline prediction. Because the age-adjusted CCI incorporates age, its coefficient should not be interpreted as directly equivalent to the ECI coefficient. The ASA PS Classification has the advantage of being universally available in perioperative care, whereas the CCI and ECI provide more structured comorbidity quantification [10,11,12,13,14,15,16]. The broadly similar performance of both baseline-only and exploratory hospital-course models does not establish superiority of any single index. Rather, clinicians and researchers may select an index based on data availability, ease of implementation, and the clinical or administrative context.

Although exploratory hospital-course models showed higher discrimination than baseline-only models, this should not be interpreted as improved baseline prediction, because in-hospital adverse events occurred during hospitalization and were conditional on survival time and documentation [22]. These models are therefore best viewed as descriptive hospital-course models combining baseline vulnerability with markers of postoperative clinical deterioration. Decision-curve analysis was included as an exploratory assessment of potential clinical net benefit, but it should be interpreted cautiously because the models were not externally validated and were not developed as definitive bedside prediction tools [25,26].

The association between general anesthesia and mortality in Charlson and Elixhauser models, but not in ASA PS model, should be interpreted cautiously. Anesthetic technique was not randomized and was likely influenced by patient condition, fracture characteristics, perioperative urgency, anticoagulant therapy, and clinician judgment. This finding may therefore reflect confounding by indication rather than an independent effect of anesthesia type.

Time to surgery was associated with mortality in univariable analysis, but this association was no longer observed after adjustment for other clinical factors. Although earlier surgery is generally recommended when feasible [19,27,28], time to surgery in this study was recorded in days rather than hours, which limits interpretation around commonly used 24–48 h thresholds. Longer time to surgery may also reflect poorer baseline condition or the need for medical optimization before surgery, rather than the effect of surgical delay alone.

Previous Croatian studies have highlighted hip fractures as an important clinical and public health burden in older adults, reporting increasing fracture numbers, the relevance of comorbidity and ASA classification, and national data on incidence and survival [29,30,31]. Compared with these studies, our work adds a contemporary regional cohort focused on 30-day mortality, comparative performance of ASA PS Classification, CCI, and ECI, and cautious interpretation of in-hospital adverse events.

The strengths of this study include a relatively large real-world cohort of surgically treated hip fracture patients from a regional tertiary center, complete 30-day mortality ascertainment, and a same-cohort comparison of three commonly used comorbidity indices. The study also included detailed perioperative variables and documented in-hospital adverse events, enabling evaluation of both baseline vulnerability and the entire course under the same clinical and institutional conditions.

These findings should be interpreted considering several limitations. The retrospective single-center design may have introduced selection bias, information bias, and residual confounding, particularly because frailty, functional status, cognitive impairment, nutritional status, nursing-home residence, and COVID-19 status were not consistently available. In addition, 207 patients were excluded because of incomplete clinical documentation, and no multiple imputation was performed. A formal comparison between included and excluded patients was not feasible because standardized screening-level variables were not reliably available for all excluded patients. Therefore, selection bias related to complete-case analysis cannot be excluded.

In-hospital adverse events were based on clinical documentation, exact event-onset dates were unavailable, and time-dependent Cox modelling or landmark analysis could not be performed. Consequently, we could not determine whether documented adverse events preceded the period of increased mortality risk or instead reflected ongoing clinical deterioration before death. Patients who died early may have had less opportunity to develop or have documented adverse events, which could introduce time-related bias. Surgery-related adverse events were not consistently available, and several individual adverse-event categories were sparse; therefore, component-level findings should be interpreted descriptively and with caution.

From a clinical perspective, the findings support the importance of baseline risk recognition and careful monitoring of patients undergoing hip fracture surgery. However, the present data cannot prove that preventing specific adverse events will necessarily reduce mortality. Future prospective multicenter studies incorporating frailty, functional status, cognitive impairment, nutritional status, detailed adverse event timing, and time-dependent analytical approaches are needed to improve prognostic modelling and better characterize clinical trajectories associated with short-term mortality after hip fracture surgery.

## 5. Conclusions

In this retrospective cohort of surgically treated hip fracture patients, 30-day mortality was 11.0% and was associated with older age, male sex, higher baseline comorbidity burden, and documented in-hospital adverse events. Baseline-only models, based on the ASA PS Classification, the CCI, and the ECI, showed acceptable and broadly comparable discriminative performance for 30-day mortality, suggesting that routinely available comorbidity measures provide broadly similar short-term risk stratification in this setting. In-hospital adverse events should be interpreted as time-dependent events associated with an adverse clinical trajectory and not as baseline predictors or evidence of causality. These findings support careful monitoring and early recognition of clinical deterioration, while highlighting the need for prospective studies incorporating frailty, functional status, cognitive impairment, and time-dependent analyses of adverse events.

## Figures and Tables

**Table 1:** Baseline and perioperative characteristics of the study population (*n* = 785).

Variable	*n* (%) or Median (IQR)
Age (years)	84 (78–89)
Male sex	196 (25.0)
ASA PS III-IV	436 (55.5)
CCI ≥ 5	458 (58.3)
ECI ≥ 5	367 (46.8)
Admission hemoglobin (g/L)	121 (108–133)
Any pre-admission antithrombotic therapy	256 (32.6)
Fracture type	Femoral neck 335 (42.7); pertrochanteric/subtrochanteric 432 (55.0); other 18 (2.3)
Main surgical procedures	PFNA 437 (55.7); hip hemiarthroplasty 265 (33.8)
General anesthesia	116 (14.8)
Preoperative transfusion	70 (8.9)
Time to surgery (days)	3 (1–5)

Values are presented as number (%) or median (interquartile range). ASA PS, American Society of Anesthesiologists Physical Status; CCI, Charlson Comorbidity Index; ECI, Elixhauser Comorbidity Index; IQR, interquartile range; PFNA, proximal femoral nail antirotation. Detailed categorical distributions are provided in Supplementary Table S1.

**Table 2:** Distribution of documented in-hospital adverse events and corresponding 30-day mortality rates.

Adverse Event Group	Patients, *n* (%)	Deaths, *n*	Mortality, %
Any in-hospital adverse event	190 (24.2)	52	27.4
Urinary tract infection	60 (7.6)	9	15.0
Pressure ulcer	55 (7.0)	16	29.1
Respiratory infections	56 (7.1)	20	35.7
Venous thromboembolism	14 (1.8)	3	21.4
Cardiovascular events	3 (0.4)	1	33.3
Other medical adverse events	23 (2.9)	6	26.1

Categories were not mutually exclusive; some patients experienced more than one in-hospital adverse event. Detailed components of respiratory infections, venous thromboembolism, cardiovascular events, and other medical adverse events are provided in Supplementary Table S4. Estimates for sparse categories, particularly cardiovascular events and venous thromboembolism, should be interpreted descriptively and with caution.

**Table 3:** Univariable logistic regression analysis of factors associated with 30-day mortality.

Variable	OR (95% CI)	*p*-Value
Older age category	2.12 (1.59–2.85)	<0.001
Male sex	2.56 (1.62–4.07)	<0.001
ASA PS Classification (per class increase)	3.46 (2.21–5.41)	<0.001
CCI (per point increase)	1.37 (1.23–1.52)	<0.001
ECI (per point increase)	1.09 (1.05–1.12)	<0.001
Admission hemoglobin (per g/L increase)	0.98 (0.97–0.99)	0.002
Time to surgery (per day increase)	1.12 (1.05–1.21)	0.002
Preoperative transfusion	2.23 (1.18–4.21)	0.013
General anesthesia	2.05 (1.20–3.52)	0.009
Any in-hospital adverse event	6.22 (3.88–9.95)	<0.001

Odds ratios were obtained using univariable logistic regression analysis. ASA PS Classification, American Society of Anesthesiologists Physical Status Classification System; CCI, Charlson Comorbidity Index; CI, confidence interval; ECI, Elixhauser Comorbidity Index; OR, odds ratio.

**Table 4:** Baseline-only multivariable logistic regression models for factors associated with 30-day mortality.

Variable	OR (95% CI)	*p*-Value
ASA PS baseline model		
Older age category	1.94 (1.43–2.63)	<0.001
Male sex	3.58 (2.16–5.94)	<0.001
ASA PS Classification System	2.37 (1.46–3.84)	<0.001
Admission hemoglobin	0.99 (0.98–1.00)	0.063
Charlson baseline model		
Male sex	3.14 (1.93–5.13)	<0.001
Age-adjusted CCI	1.34 (1.20–1.50)	<0.001
Admission hemoglobin	0.98 (0.97–1.00)	0.008
Elixhauser baseline model		
Older age category	2.22 (1.64–3.01)	<0.001
Male sex	3.59 (2.18–5.91)	<0.001
ECI	1.07 (1.04–1.11)	<0.001

Baseline-only models excluded in-hospital adverse events and were used for baseline comorbidity model comparison. The ASA PS baseline model included age category, sex, ASA PS Classification, and admission hemoglobin; the Charlson baseline model included sex, age-adjusted CCI, and admission hemoglobin; and the Elixhauser baseline model included age category, sex, and ECI. Separate models were constructed to avoid collinearity and conceptual overlap. CI, confidence interval; OR, odds ratio; ASA PS, American Society of Anesthesiologists Physical Status; CCI, Charlson Comorbidity Index; ECI, Elixhauser Comorbidity Index.

**Table 5:** Exploratory hospital-course multivariable logistic regression models for factors associated with 30-day mortality.

Variable	OR (95% CI)	*p*-Value
**ASA PS model**		
Older age category	2.01 (1.46–2.76)	<0.001
Male sex	3.43 (2.00–5.88)	<0.001
ASA PS Classification System	2.11 (1.27–3.52)	0.004
Any in-hospital adverse event	4.86 (2.90–8.15)	<0.001
Admission hemoglobin	0.99 (0.98–1.01)	0.270
Time to surgery	1.03 (0.95–1.12)	0.418
Preoperative transfusion	0.68 (0.27–1.69)	0.403
General anesthesia	1.66 (0.90–3.05)	0.106
**Charlson model**		
Male sex	2.95 (1.75–4.97)	<0.001
Age-adjusted CCI	1.33 (1.18–1.50)	<0.001
Any in-hospital adverse event	4.93 (2.96–8.22)	<0.001
Admission hemoglobin	0.99 (0.98–1.01)	0.206
Time to surgery	1.04 (0.96–1.12)	0.387
Preoperative transfusion	0.77 (0.31–1.93)	0.580
General anesthesia	1.84 (1.02–3.33)	0.043
**Elixhauser model**		
Older age category	2.14 (1.56–2.94)	<0.001
Male sex	3.56 (2.09–6.05)	<0.001
ECI	1.07 (1.03–1.11)	<0.001
Any in-hospital adverse event	4.89 (2.92–8.17)	<0.001
Time to surgery	1.04 (0.95–1.13)	0.415
Preoperative transfusion	1.03 (0.48–2.19)	0.944
General anesthesia	1.90 (1.05–3.46)	0.035

Separate models were constructed to avoid collinearity and conceptual overlap. These models include in-hospital adverse events and should therefore be interpreted as exploratory hospital-course models rather than baseline prediction models. The in-hospital adverse event variable represents a time-dependent hospital-course event associated with mortality, not a baseline predictor or evidence of causality. CI, confidence interval; OR, odds ratio.

**Table 6:** Model performance of baseline-only and exploratory hospital-course models.

Model	AUC (Bootstrap 95% CI)	Hosmer–Lemeshow *p*-Value	Brier Score
Baseline-only ASA PS model	0.76 (0.71–0.81)	0.453	0.085
Baseline-only Charlson model	0.73 (0.67–0.78)	0.693	0.089
Baseline-only Elixhauser model	0.77 (0.71–0.82)	0.144	0.087
Exploratory ASA PS hospital-course model	0.82 (0.77–0.87)	0.894	0.077
Exploratory Charlson hospital-course model	0.80 (0.75–0.85)	0.687	0.081
Exploratory Elixhauser hospital-course model	0.82 (0.77–0.86)	0.401	0.077

AUC, area under the receiver operating characteristic curve; ASA PS, American Society of Anesthesiologists Physical Status. Baseline-only models excluded in-hospital adverse events and were used for baseline comorbidity model comparison. Exploratory hospital-course models included documented in-hospital adverse events and should not be interpreted as baseline prediction models.

**Table 7:** Thirty-day mortality according to number of documented in-hospital adverse events.

Number of Events	Patients, *n* (%)	Deaths, *n*	Mortality, %	*p*-Value
0	595 (75.8)	34	5.7	<0.001
1	157 (20.0)	38	24.2	
≥2	33 (4.2)	14	42.4	

Values are presented as numbers (*n*) and percentages (%). The *p*-value was calculated using the chi-square test.

## Data Availability

The datasets generated and analyzed during the current study are not publicly available due to ethical and privacy restrictions but are available from the corresponding author on reasonable request and subject to institutional approval.

## Supplementary Materials

The following supporting information can be downloaded at: https://www.mdpi.com/article/10.3390/healthcare14131870/s1. Table S1: Detailed baseline and perioperative characteristics of the study population (*n* = 785). Table S2: Baseline and perioperative characteristics according to 30-day mortality status. Table S3: Continuous clinical variables according to 30-day mortality status. Table S4: Detailed components of in-hospital adverse events. Table S5: Sensitivity analysis of time to surgery categories and 30-day mortality.

## Abbreviations

The following abbreviations are used in this manuscript:

ASA PS Classification	American Society of Anesthesiologists Physical Status Classification System
AUC	Area under the receiver operating characteristic curve
CCI	Charlson Comorbidity Index
CI	Confidence interval
COVID-19	Coronavirus disease 2019
ECI	Elixhauser Comorbidity Index
HIS	Hospital information system
ICD-10	International Classification of Diseases, 10th Revision
ICU	Intensive care unit
IQR	Interquartile range
DOAC	Direct oral anticoagulants
OR	Odds ratio
PFNA	Proximal femoral nail antirotation
ROC	Receiver operating characteristic
SPSS	Statistical Package for the Social Sciences
STROBE	Strengthening the Reporting of Observational Studies in Epidemiology
