# Supplementary material for: Thirty-Day Mortality After Hip Fracture Surgery: Association with In-Hospital Adverse Events and Comparative Performance of Comorbidity Indices
Source: Healthcare (Basel). 2026 Jun 26;14(13):1870. doi: 10.3390/healthcare14131870 (PMC13362407; doi:10.3390/healthcare14131870)
Supplement: Supplementary file 1 [file healthcare-14-01870-s001.zip › healthcare-4331509-supplementary.pdf]

healthcare

MDPI

Supplementary Materials

Manuscript title: 30-Day Mortality After Hip Fracture Surgery: Association With In-Hospital Adverse Events and Comparative Performance of Comorbidity Indices

Table S1. Detailed baseline and perioperative characteristics of the study population (n = 785).

Variable	n (%) or median (IQR)
Age (years)	84 (78-89)
Sex	
Male	196 (25.0)
Female	589 (75.0)
ASA PS Classification System	
ASA PS I	15 (1.9)
ASA PS II	334 (42.5)
ASA PS III	416 (53.0)
ASA PS IV	20 (2.5)
CCI	
0	8 (1.0)
1-2	66 (8.4)
3-4	253 (32.2)
>=5	458 (58.3)
ECI	
<0	35 (4.5)
0	237 (30.2)
1-4	146 (18.6)
>=5	367 (46.8)
Admission hemoglobin (g/L)	121 (108-133)
Admission hematocrit (L/L)	0.36 (0.32-0.39)
Pre-admission antithrombotic therapy	None 529 (67.4); acetylsalicylic acid 117 (14.9); warfarin 27 (3.4); DOAC 86 (11.0); other 26 (3.3)
Fracture type	
Femoral neck fracture	335 (42.7)
Pertrochanteric fracture	374 (47.6)
Subtrochanteric fracture	58 (7.4)
Other	18 (2.3)
Surgical procedure	
PFNA	437 (55.7)
Hip hemiarthroplasty	265 (33.8)
Total hip arthroplasty	32 (4.1)
Other procedures	51 (6.5)
Type of anesthesia	
Spinal anesthesia	669 (85.2)
General anesthesia	116 (14.8)
Perioperative transfusion	Preoperative 70 (8.9); intraoperative 64 (8.2); postoperative 257 (32.7)
Time to surgery (days)	3 (1-5)

Values are presented as number (%) or median (interquartile range). ASA PS, American Society of Anesthesiologists Physical Status; CCI, Charlson Comorbidity Index; DOAC, direct oral anticoagulants; ECI, Elixhauser Comorbidity Index; Hb, hemoglobin; Hct, hematocrit; IQR, interquartile range; PFNA, proximal femoral nail antirotation; TTS, time to surgery.

Table S2. Baseline and perioperative characteristics according to 30-day mortality status.

Variable	Survivors (n = 699)	Non-survivors (n = 86)	p-value
Age category			
>=90 years	63 (9.0)	18 (20.9)	<0.001
80-89 years	321 (45.9)	54 (62.8)	
70-79 years	181 (25.9)	10 (11.6)	
60-69 years	80 (11.4)	3 (3.5)	
50-59 years	38 (5.4)	1 (1.2)	
<50 years	16 (2.3)	0 (0.0)	
Sex			
Male	159 (22.7)	37 (43.0)	<0.001
Female	540 (77.3)	49 (57.0)	
ASA PS Classification System			
ASA PS I	15 (2.1)	0 (0.0)	<0.001
ASA PS II	317 (45.4)	17 (19.8)	
ASA PS III	355 (50.8)	61 (70.9)	
ASA PS IV	12 (1.7)	8 (9.3)	
CCI category			
0	7 (1.0)	1 (1.2)	<0.001
1-2	66 (9.4)	0 (0.0)	
3-4	241 (34.5)	12 (14.0)	
>=5	385 (55.1)	73 (84.9)	
ECI category			
<0	31 (4.4)	4 (4.7)	<0.001
0	221 (31.6)	16 (18.6)	
1-4	139 (19.9)	7 (8.1)	
>=5	308 (44.1)	59 (68.6)	
Pre-admission antithrombotic therapy			
None	478 (68.4)	51 (59.3)	0.075
Acetylsalicylic acid	106 (15.2)	11 (12.8)	
Warfarin	23 (3.3)	4 (4.7)	
DOAC	69 (9.9)	17 (19.8)	
Other	23 (3.3)	3 (3.5)	
Fracture type			
Femoral neck fracture	301 (43.1)	34 (39.5)	0.938
Pertrochanteric fracture	331 (47.4)	43 (50.0)	
Subtrochanteric fracture	51 (7.3)	7 (8.1)	
Other	16 (2.3)	2 (2.3)	
Surgical procedure			
PFNA	387 (55.4)	50 (58.1)	0.711
Hip hemiarthroplasty	235 (33.6)	30 (34.9)	
Total hip arthroplasty	30 (4.3)	2 (2.3)	
Other procedures	47 (6.7)	4 (4.7)	
Type of anesthesia			
Spinal anesthesia	604 (86.4)	65 (75.6)	0.012
General anesthesia	95 (13.6)	21 (24.4)	
Preoperative transfusion			

No	643 (92.0)	72 (83.7)	0.019
Yes	56 (8.0)	14 (16.3)	
Intraoperative transfusion			
No	643 (92.0)	78 (90.7)	0.838
Yes	56 (8.0)	8 (9.3)	
Postoperative transfusion			
No	475 (68.0)	53 (61.6)	0.290
Yes	224 (32.0)	33 (38.4)	

Values are presented as n (%). p-values were calculated using the chi-square test. Age categories were analyzed as source-coded decade-based groups used in the dataset. CCI, Charlson Comorbidity Index; ECI, Elixhauser Comorbidity Index.

Table S3. Continuous clinical variables according to 30-day mortality status.

Variable	Survivors (n = 699), median (IQR)	Non-survivors (n = 86), median (IQR)	p-value
Admission hemoglobin (g/L)	122 (109-134)	114 (102-128)	0.001
Admission hematocrit (L/L)	0.36 (0.32-0.39)	0.34 (0.31-0.38)	0.011
Time to surgery (days)	3 (1-4)	4 (2-5)	0.005
ICU length of stay (days)	0 (0-1)	1 (0-3)	<0.001
Total hospital length of stay (days)	10 (8-12)	10 (8-14)	0.544

p-values were calculated using the Mann-Whitney U test. ICU, intensive care unit; IQR, interquartile range.

Table S4. Detailed components of in-hospital adverse events.

Adverse event group	Component	Patients, n (%)	Deaths, n	Mortality, %
Overall	Any in-hospital adverse event	190 (24.2)	52	27.4
Respiratory infections	Respiratory infections overall	56 (7.1)	20	35.7
	Pneumonia	34 (4.3)	13	38.2
	COVID-19 infection	22 (2.8)	7	31.8
Urinary tract infection	Urinary tract infection	60 (7.6)	9	15.0
Pressure ulcer	Pressure ulcer	55 (7.0)	16	29.1
Venous thromboembolism	Venous thromboembolism overall	14 (1.8)	3	21.4
	Deep vein thrombosis	10 (1.3)	3	30.0
	Pulmonary embolism	3 (0.4)	0	0.0
	Combined, other venous thromboembolism	1 (0.1)	0	0.0
Cardiovascular events	Cardiovascular events overall	3 (0.4)	1	33.3
	Atrioventricular block/pacemaker	1 (0.1)	1	100.0
	Atrial fibrillation	1 (0.1)	0	0.0
	Postoperative tachycardia	1 (0.1)	0	0.0
Other medical adverse events	Other medical adverse events overall	23 (2.9)	6	26.1
	Acute kidney injury	15 (1.9)	5	33.3
	Neurological complication / POCD	8 (1.0)	1	12.5

Rows are not mutually exclusive; some patients experienced more than one in-hospital adverse event. Component rows are descriptive and clarify the composition of broader adverse-event groups. Exact dates of event onset were not consistently available; therefore, these component-level results should not be interpreted as time-dependent effects.

Table S5. Sensitivity analysis of time to surgery categories and 30-day mortality.

Time to surgery category	Patients, n (%)	Deaths, n	Mortality, %	p-value
0-1 days	213 (27.1)	18	8.5	0.012
2 days	138 (17.6)	8	5.8	
>2 days	434 (55.3)	60	13.8	

p-value was calculated using the chi-square test. Time to surgery was recorded in days because exact times in hours were not consistently available in the retrospective documentation.
